# Nutritional Support for Pediatric Severe Traumatic Brain Injury

**DOI:** 10.3389/fped.2022.904654

**Published:** 2022-05-17

**Authors:** Elizabeth Elliott, Michael Shoykhet, Michael J. Bell, Kitman Wai

**Affiliations:** Critical Care Medicine, Children's National Hospital, Washington, DC, United States

**Keywords:** nutrition, traumatic brain injury, pediatric intensive care unit, neurocritical care, enteral nutrition

## Abstract

In critically ill children with severe traumatic brain injury (sTBI), nutrition may help facilitate optimal recovery. There is ongoing research regarding nutritional practices in the pediatric intensive care unit (PICU). These are focused on identifying a patient's most appropriate energy goal, the mode and timing of nutrient delivery that results in improved outcomes, as well as balancing these goals against inherent risks associated with nutrition therapy. Within the PICU population, children with sTBI experience complex physiologic derangements in the acute post-injury period that may alter metabolic demand, leading to nutritional needs that may differ from those in other critically ill patients. Currently, there are relatively few studies examining nutrition practices in PICU patients, and even fewer studies that focus on pediatric sTBI patients. Available data suggest that contemporary neurocritical care practices may largely blunt the expected hypermetabolic state after sTBI, and that early enteral nutrition may be associated with lower morbidity and mortality. In concordance with these data, the most recent guidelines for the management of pediatric sTBI released by the Brain Trauma Foundation recommend initiation of enteral nutrition within 72 h to improve outcome (Level 3 evidence). In this review, we will summarize available literature on nutrition therapy for children with sTBI and identify gaps for future research.

## Introduction

Nutritional support is an important component of the comprehensive care for critically ill children. Evidence shows that appropriate delivery of energy and macronutrients maximizes the potential for positive outcomes in the pediatric intensive care unit (PICU), while over- or underfeeding may place patients at risk for increased morbidity and mortality (1–9). Guidelines from the Society of Critical Care Medicine (SCCM) and American Society for Parenteral and Enteral Nutrition (ASPEN) suggest using indirect calorimetry (IC) to determine an appropriate caloric goal, initiating enteral nutrition (EN) within 24–48 h of PICU admission, and reaching two-thirds of the caloric goal by 7 days (10, 11).

However, questions remain about the optimal prescription of nutrition for children that have suffered a severe traumatic brain injury (sTBI). Prescribing nutrition therapy that is targeted for this population is important because inadequate nutrition results in loss of lean body mass and reduced functional capacity, both of which are uniquely important for recovery from sTBI (12, 13). Additionally, the pathophysiology underlying secondary injury cascades could be modulated through nutritional targets (14).

Here we will present available data regarding nutrition for pediatric patients in the acute period after sTBI, the potential impact of nutrition on patient outcome, and identify gaps for future research.

## Energy Expenditure After TBI

In the PICU, nutrition support should be individualized to each patient's unique needs because both overfeeding and underfeeding are associated with deleterious effects (15, 16). However, estimating energy and protein requirements is a complex task. In healthy children, nutritional needs vary greatly depending on age, growth velocity, body composition, current nutritional status, and level of physical activity (17). When children become critically ill, the number of variables grows to include fever, sepsis, sedative/neuromuscular blockade, pharmacotherapy, and duration and stage of critical illness (18). Predictive equations are frequently used to estimate energy expenditure and guide prescription of nutrition in critically ill children, but they may over- or underestimate the true metabolic demand (18). The gold standard for measuring resting energy expenditure is indirect calorimetry.

Several studies have applied this technique to sTBI patients to determine their energy expenditure in the acute post-injury period. Original data from several decades ago suggested that sTBI induced a state of hypermetabolism with energy expenditure up to 170% of expected (19, 20). However, these studies were performed before contemporary neurocritical care practices were firmly established, and it has been shown that neuroprotective strategies such as temperature control (21), sedation (22), neuromuscular blockade (23), and burst suppression (24) result in decreased energy expenditure in sTBI patients. In support of this notion, recent studies of energy expenditure after sTBI do not show the same degree of hypermetabolism, with some studies even showing hypometabolism during the acute post-injury period (25, 26). In a prospective observational study by Mtaweh et al. (27), 13 pediatric patients in their first week after sTBI had intermittent measurements of energy expenditure (MEE) by indirect calorimetry. All patients were intubated and sedated with opiates, nearly all were receiving neuromuscular blockade, and none were febrile or seizing during testing. MEE was then compared to predicted resting energy expenditure (pREE) as calculated by two commonly used predictive equations: the Harris-Benedict equation and the Schofield equation. The result was an average MEE/pREE ratio of 70.2 ± 3.8% and 69 ± 4.5% expected, respectively.

These data support the notion that contemporary neurocritical care practices may be acting to blunt any expected hypermetabolism. This results in a hypometabolic state during the first week post-injury. Because of this difficulty in predicting energy expenditure after sTBI, indirect calorimetry remains the gold standard for determining caloric need and should be used whenever feasible.

## Mode of Nutrition Delivery

In addition to determining total energy requirement, clinicians must decide the safest and most appropriate mode of nutrient delivery. EN is increasingly considered as the preferred mode of delivery. Compared to parental nutrition (PN), EN may decrease the risk of metabolic derangements, hepatobiliary dysfunction, and infection. EN is also considerably less costly compared to PN (28, 29). In a multicenter, randomized controlled trial of 1,440 critically ill pediatric patients, Fivez et al. showed that delaying the initiation of PN until the 8^th^ day of hospitalization was superior to initiation of PN within 24 h of admission. Patients with delayed initiation of PN had lower rates of new infections, shorter durations of mechanical ventilatory support, and shorter durations of PICU stay (30). A similar result was seen in a large international study of nutrient delivery in mechanically ventilated children by Mehta et al. The study found that intake of a higher percentage of the prescribed dietary energy goal enterally is associated with improved 60-day survival, while mortality is higher in patients who received any amount of PN compared to those that received none (5).

With regard to studies specifically examining TBI patients, a small, randomized control trial of adult patients with moderate TBI by Meirelles et al. showed no difference in mortality, length of ICU stay, and days of mechanical ventilation based on mode of energy delivery (31). However, the authors did report significantly higher serum glucose in the PN group compared to the EN group (102.4 vs. 133.3 mg/dL). To our knowledge, there are no studies evaluating mode of nutrient delivery in pediatric sTBI.

## Timing of Support

Delays in nutrition initiation and frequent interruptions to feeding commonly result in largely insufficient macronutrient delivery in PICU patients (5). Numerous studies have shown benefit for patients who receive early EN and for those who achieve adequate energy and protein delivery within 7–10 days (3, 5, 6, 8, 32). Although there is evidence that early EN is feasible and safe in the PICU (33–35), sTBI patients experience many barriers to reaching EN goals, including but not limited to need for vasoactives, high doses of sedatives, and fasting for procedures (36).

In a large adult retrospective cohort study with sTBI patients by Härtl et al. there was significantly higher two-week mortality among patients who were not fed within 5–7 days after TBI. This result represented a 2- and 4-fold increased likelihood of death respectively compared to those who were fed within 5 days of injury (37). In addition, every 10-kcal/kg decrease in caloric intake over the first 5 days was associated with a 30–40% increase in mortality rates.

Similar data have been reported for pediatric TBI patients. In a retrospective analysis of 109 children aged 8–19 years with sTBI, starting nutritional support within 72 h of admission and achieving goal caloric intake by day 7 was correlated with shorter ICU length of stay and improved clinical outcome at discharge (38). Similarly, Meinert et al. showed that patients who had nutritional support initiated within 72 h post-injury had lower mortality and improved functional outcome (favorable GOS-E Peds at 6 months and 12 months) when compared to those who received no nutritional support over the first 7 days post-injury (39). And most recently, a retrospective multicenter study of pediatric TBI patients of all severities showed that initiation of EN later than 48 h after admission was correlated with worse functional status at ICU discharge (change in POPC and change in PCPC) (40). Of note, this correlation did not hold when patients were stratified by TBI severity.

## Site of Enteral Nutrition Delivery

Feeding intolerance, though hard to define, is common in critically ill children. PICU patients that are unable to tolerate gastric feeding may receive EN that is delivered to the small bowel via naso-duodenal or naso-jejunal feeding tube. A randomized controlled trial of pediatric patients receiving mechanical ventilation showed that the percentage of daily caloric goal achieved was less in patients receiving gastric EN vs. small-bowel EN (30 ± 23% vs. 47 ± 22%) (42). There was no significant difference in signs or symptoms of feeding intolerance such as abdominal distension, vomiting, or diarrhea between groups.

sTBI patients may be more susceptible to feeding intolerance due to altered gastric motility, prolonged immobilization, and medication-related ileus (43). Due to this concern, one study in adult patients examined the tolerability of bolus vs. continuous gastric feeds and found that the patients receiving continuous feeds achieved their nutritional goals faster and had less frequent feeding intolerance (44). There are currently no pediatric studies comparing sites of EN delivery in sTBI patients.

## Immunonutrition

Given the complex interplay of the intestines with the immune system, there is emerging interest in the effect of diet beyond its strictly nutritional value (45). The acute period after sTBI is characterized by neuroinflammation that paradoxically perpetuates secondary injury while also promoting repair (46). Due to their roles in cellular metabolism and the inflammatory cascade, substances such as amino acids, electrolytes, and antioxidants have been proposed as dietary supplements that could aid in modulating this delicate balance (47). In a randomized controlled trial with pediatric sTBI patients, patients who were given an immune-enhancing diet supplemented with glutamine, arginine, antioxidants, and omega-3 fatty acids showed no difference in outcomes when compared to patients who were on a regular formula regimen (41).

Based on emerging knowledge about the gut-brain axis, it has been hypothesized that alterations in gut microbiota could influence post-traumatic neuroinflammation (48). A recent study of children with sTBI by Rogers et al. characterized the temporal and spatial alterations in the microbiome during their initial ICU admission (49). Compared to healthy controls, sTBI patients quickly developed dysbiosis. They showed depletion of beneficial bacterial species and enrichment of pathogenic bacterial species over time. Additional studies are needed to determine how these changes impact clinical outcomes.

## Glycemic Control

Hyperglycemia is a commonly observed response to the stress of critical illness, which is mediated by alterations in glucose metabolism that are caused by increases in pro-inflammatory and counter-regulatory hormones, such as cortisol and epinephrine (50). This stress hyperglycemia (SH) represents a common secondary insult to the brain after sTBI (51), and has been associated with increased morbidity and mortality in adult and pediatric patients (52–56). Studies examining the effect of tight glycemic control in PICU patients have yielded inconsistent results, with most studies showing no benefit or concern for harm from severe hypoglycemia (57–59).

In a large international RCT in adults with TBI, patients receiving intensive glucose control (target range 80–110 mg/dL) and patients receiving conventional glucose control (target of less than 180 mg/dL) had similar long-term outcomes (60). However, intensive glucose control resulted in more frequent episodes of moderate to severe hypoglycemia, suggesting possible harm. In addition, several small studies in adult sTBI patients have used cerebral microdialysis to demonstrate tight glycemic control is associated with critical reduction in glucose and elevation of lactate/pyruvate ratio in the brain (61, 62). This increase in markers of cellular distress suggests that tight glycemic control results in damaged brain tissue being unable to access the glucose that is necessary for cellular repair and survival (62). There have been no studies evaluating glucose control in pediatric sTBI patients.

## Discussion

Adequate nutrition is essential for all critically ill children because malnutrition contributes to immune dysfunction and infections (63), weakness (64), and delayed healing and recovery (65). However, delivering nutrition to patients with sTBI is not a straightforward task. Nutrition therapies for sTBI patients must take into account hydration and electrolyte goals to prevent fluid shifts that could worsen cerebral edema (66). In addition, sTBI is accompanied by unique physiologic derangements. Damage to axons in the autonomic nervous system can lead to delayed gastric emptying and intestinal hypomotility (43). These symptoms are compounded by the use of sedatives and barbiturates (67), which lead to feeding intolerance and the subsequent withholding of enteral nutrition.

Despite these factors, few studies have attempted to identify nutritional interventions that could improve outcome for this patient population (Table 1). In general, high-quality nutrition studies are scarce and studies that focus on sTBI are even more uncommon. Most studies to date have been limited by small sample size, retrospective or observational design, and inconsistent inclusion or exclusion criteria. This leads to reduced statistical power, poor generalizability, and difficulty in assessing correlation vs. causation.

**Table 1:** Nutrition for pediatric severe traumatic brain injury.

**Reference**	**Study design**	**Population**			**Study aim**	**Results/conclusions**
		**N**	Inclusion criteria^*^	Exclusion criteria^*^		
Balakrishnan et al. (40)	Retrospective	Total N = 416 (N with GCS <9 = 107)	-Age 0 to 18 -Head injury associated ICD9 diagnosis code	-Head abbreviated ISS of <2 -Died <48h from admission	To compare patients who received enteral nutrition early (≤48h) and late (>48h)	-Lower GCS and higher ISS were associated with delayed initiation of enteral nutrition -Delayed enteral nutrition was associated with worse functional status (POPC/PCPC) at PICU discharge (*p =* 0.02), but not mortality or increased length of stay
Meinert et al. (39)	Secondary analysis of RCT	N = 90	-Age 0 to 18 -Post-resuscitation GCS <9	-Normal head CT -GCS = 3 with unreactive pupils -Hypotension for >10 min -Hypoxia for >30 min -Penetrating injury	To understand the relationship between the timing of initiation of nutritional support in children with severe traumatic brain injury (TBI) and outcomes	Initiation of nutritional support before 72 hours after TBI was associated with decreased mortality (*p =* 0.01) and favorable outcome (GOS-E Peds) at 6 months (*p =* 0.03) and 12 months (*p =* 0.04)
Mtaweh et al. (27)	Prospective observational study	N = 13	*Same as Meinert et al. (39)*-Weight > 10 kg -Mechanically ventilated	*Same as Meinert et al. (39)*	To evaluate energy expenditure in a cohort of children with sTBI	-MEE/pREE averaged 70.2 ± 3.8%, suggesting that contemporary neurocritical care practices may blunt a hypermetabolic response -Mean MEE/pREE in the favorable outcome group was 76.4 ± 6% vs. 64.7 ± 4.7% in the unfavorable outcome group (*p* = 0.13)
Malakouti et al. (68)	Retrospective	sTBI N = 101 control N = 92	-Age 0 to 15 -GCS < 9	-Admitted to PICU for <7 days	To examine nutritional support in severe pediatric traumatic brain injury patients (cases) and non-traumatic brain injury patients (controls) at a single center	-Nutrition was started 53 ± 20 hrs (range 12–162) after PICU admission -Patients whose intake met nutritional goals on PICU day 7 had earlier initiation of nutrition support than patients who never met the goals; both *p* < 0.001 -Average caloric and protein intakes for PICU days 0-7 were less for the TBI group (52% ± 16% caloric goal and 42% ± 18% protein goal) than for the non-traumatic brain injury group (65% ± 11% of caloric goal and 51% ± 20% of protein goal); both *p* < 0.01
Taha et al. (38)	Retrospective	N = 109	-Age 8 to 18 -GCS < 9	-Multisystem trauma	To examine the timing of nutritional supplement initiation and the timing of achieving full caloric intake in pediatric sTBI	-Starting nutritional support within 72 h of and admission and achieving goal caloric intake by day 7 was correlated with shorter ICU length of stay (*p* < 0.01) and improved clinical outcome (modified PCPC) at discharge (*p* < 0.05)
Briassoulis et al. (41)	RCT	*N =* 40 (*n* = 20 for immune-enhancing diet, n = 20 for standard enteral diet)	-GCS <9 -Age 0 to 18 6 -Enteral feeds starting within 12h of admission	-Renal or gastrointestinal disease	To analyze the effect of an immune enhancing (IE) diet on infection and metabolic indices in children with sTBI	-Interleukin-8 levels were lower in the IE group compared with the regular formula group by day 5 (*p* < 0.04) -Less gastric cultures were positive in the IE group compared with the regular formula group (26.7 vs. 71.4%; *p* < 0.02) -Nosocomial infections (15 vs. 25%), length of stay (16.7 vs. 12.2 days), length of mechanical ventilation (11 vs. 8 days), and survival (80 vs. 95%) did not differ between groups
Havalad et al. (26)	Retrospective	*N =* 30	-GCS < 9 -Mechanically ventilated	-Required inotropes or pentobarbital	To determine if pREE varies significantly from MEE in a population of head-injured children	-More than half of the estimates of REE differed from measured REE by >10% and there was no correlation between severity of illness and measured REE to explain these inaccuracies, which suggests that nutrition should be prescribed to children with sTBI based on REE to avoid consequences of overfeeding or malnutrition

* *Selected criteria relevant for understanding sample characteristics. TBI, traumatic brain injury; sTBI, severe TBI; ISS, illness severity score; GCS, glascow coma scale; MEE, measured energy expenditure; pREE, predicted resting energy expenditure*.

In 2019, the Brain Trauma Foundation released updated guidelines for the management of pediatric sTBI, which include a section on nutrition (69). Based on available evidence the authors were able to make two primary recommendations: (1) early initiation of EN (within 72 h of injury) to decrease mortality and improve outcomes, and (2) immune-modulating diet is not recommended to improve outcomes. These recommendations are largely consistent with established guidelines for all critically ill pediatric patients. The currently available data are insufficient to warrant making further statements about nutritional goals that are unique to sTBI patients; providers should continue to prescribe nutritional support according to the previously mentioned SCCM/ASPEN guidelines.

Further studies are needed to elucidate nutritional recommendations for sTBI patients. It is important that future studies are designed in a manner that generates high-quality data. These studies should optimally be large, prospective trials, and should seek to identify unique nutrition targets for pediatric sTBI patients. This includes identifying an appropriate caloric goal and identifying a threshold for the initiation of targeted glucose control. Additional studies should also seek answers to questions about the timing, mode, and composition of macronutrient delivery and their effect on outcome.

Beyond the strictly caloric value of a sTBI patient's diet, nutritional supplementation may present an opportunity to mediate the effects of brain injury on a cellular level (14, 47, 66). Future clinical and pre-clinical studies should examine how supplementation of certain electrolytes, vitamins, minerals, or amino acids could interact with cellular processes to mediate the inflammatory cascade and restore normal metabolic activities.

Another important consideration when designing future studies is the choice of outcome measure. Although mortality is a traditional primary outcome, functional status is equally important and may be more pertinent in pediatric research (70). Future studies should examine how nutrition combined with early mobilization and rehabilitation impacts deconditioning, muscle wasting, and change in overall functional status. Completion of high-quality studies that answer these questions will maximize the potential for positive outcomes in survivors of sTBI.

Of note, the Approaches and Decisions for Acute Pediatric TBI (ADAPT) Trial recently completed enrollment of 1,000 pediatric patients with sTBI. This trial was designed to use comparative effectiveness to examine the acute medical management of infants, children, and adolescents with severe TBI. Several of the a priori hypotheses put forward by the ADAPT investigators addressed nutrition questions, including many of those raised in this article. We anticipate new data will emerge from ADAPT and from other innovative trial designs, and we look forward to additional studies that will allow for more robust recommendations in the future.

## Author Contributions

EE, MS, MB, and KW contributed to the writing and editing of this manuscript. All authors contributed to the article and approved the submitted version.

## Conflict of Interest

The authors declare that the research was conducted in the absence of any commercial or financial relationships that could be construed as a potential conflict of interest.

## Publisher's Note

All claims expressed in this article are solely those of the authors and do not necessarily represent those of their affiliated organizations, or those of the publisher, the editors and the reviewers. Any product that may be evaluated in this article, or claim that may be made by its manufacturer, is not guaranteed or endorsed by the publisher.

## References

[1] Jacobs A Verlinden I Vanhorebeek I van den Berghe G. Early supplemental parenteral nutrition in critically ill children: An update. Journal of Clinical Medicine. (2019) 8:7–9. 10.3390/jcm8060830 31212639 PMC6616588

[2] Iyer R Bansal A. What do we know about optimal nutritional strategies in children with pediatric acute respiratory distress syndrome? Ann Transl Med. (2019) 7:510. 10.21037/atm.2019.08.25 31728363 PMC6828788

[3] Bechard LJ Staffa SJ Zurakowski D Mehta NM. Time to achieve delivery of nutrition targets is associated with clinical outcomes in critically ill children. Am J Clin Nutr. (2021) 114:1859–67. 10.1093/ajcn/nqab244 34320161

[4] Mehta NM Bechard LJ Dolan M Ariagno K Jiang H Duggan C. Energy imbalance and the risk of overfeeding in critically ill children. Pediat Crit Care Med. (2011) 12:398–405. 10.1097/PCC.0b013e3181fe279c 20975614 PMC4151116

[5] Mehta N Bechard L Cahill N Wang M Day A Duggan C. Nutritional practices and their relationship to clinical outcomes in critically ill children—An international multicenter cohort study. Crit Care Med. (2012) 40:2204–11. 10.1097/CCM.0b013e31824e18a8 22564954 PMC3704225

[6] Srinivasan V Hasbani N Mehta N Irving S Kandil S Allen HC. Early enteral nutrition is associated with improved clinical outcomes in critically ill children: a secondary analysis of nutrition support in the HALF-PINT trial. Pediatr Crit Care Med. (2020) 21:213–21. 10.1097/PCC.0000000000002135 31577692 PMC7060827

[7] Wong JJM Han WM Sultana R Loh TF Lee JH. Nutrition delivery affects outcomes in pediatric acute respiratory distress syndrome. J Parenteral Enteral Nutr. (2017) 41:1007–13. 10.1177/0148607116637937 26962064

[8] Mikhailov TA Kuhn EM Manzi J Christensen M Collins M Brown AM. Early enteral nutrition is associated with lower mortality in critically ill children. J Parenteral Enteral Nutr. (2014) 38:459–66. 10.1177/0148607113517903 24403379

[9] Neef M de Geukers VGM Dral A Lindeboom R Sauerwein HP Bos AP. Nutritional goals, prescription and delivery in a pediatric intensive care unit. Clin Nutr. (2008) 27:65–71. 10.1016/j.clnu.2007.10.013 18068875

[10] Mehta NM Skillman HE Irving SY Coss-bu JA Vermilyea S Farrington EA. Guidelines for the provision and assessment of nutrition support therapy in the pediatric critically ill patient : society of critical care medicine and american society for parenteral and enteral nutrition. J Parenteral Enteral Nutr. (2017) 41:706–42. 10.1177/0148607117711387 28686844

[11] Framson CMH LeLeiko NS Dallal GE Roubenoff R Snelling LK Dwyer J. Energy expenditure in critically ill children. Pediat Crit Care Med. (2007) 8:264–7. 10.1097/01.PCC.0000262802.81164.03 17417117

[12] Zhu XL Poon WS Chan CCH Chan SSH. Does intensive rehabilitation improve the functional outcome of patients with traumatic brain injury (TBI)? A randomized controlled trial. Brain Injury. (2007) 21:681–90. 10.1080/02699050701468941 17653942

[13] Skillman H Zebuhr C. Optimal Nutrition for Acute Rehabilitation in the PICU. J Pediatr Intens Care. (2015) 04:194–203. 10.1055/s-0035-1563546 31110872 PMC6513169

[14] Lucke-Wold BP Logsdon AF Nguyen L Eltanahay A Turner RC Bonasso P. Supplements, nutrition, and alternative therapies for the treatment of traumatic brain injury. Nutr Neurosci. (2018) 21:79–91. 10.1080/1028415X.2016.1236174 27705610 PMC5491366

[15] McClave SA. Indirect calorimetry: relevance to patient outcome. Respir Care Clin N Am. (2006) 12:635–50. 10.1016/j.rcc.2006.09.008 17150436

[16] Chwals WJ. Overfeeding the critically ill child: fact or fantasy? New Horiz. (1994) 2:147–55. 7922439

[17] Pontzer H Yamada Y Sagayama H Ainslie PN Andersen LF Anderson LJ. Daily energy expenditure through the human life course. Science. (2021) 373:808–12. 10.1126/science.abe5017 34385400 PMC8370708

[18] Larsen BMK Beggs MR Leong AY Kang SH Persad R Garcia Guerra G. Can energy intake alter clinical and hospital outcomes in PICU? Clin Nutr ESPEN. (2018) 24:41–6. 10.1016/j.clnesp.2018.02.002 29576361

[19] Moore R Najarian MP Konvolinka CW. Measured energy expenditure in severe head trauma. J Trauma. (1989) 29:1633–6. 10.1097/00005373-198912000-00007 2593191

[20] Phillips R Ott L Young B Walsh J. Nutritional support and measured energy expenditure of the child and adolescent with head injury. J Neurosurg. (1987) 67:846–51. 10.3171/jns.1987.67.6.0846 3119794

[21] Bruder N Raynal M Pellissier D Courtinat C Francois G. Influence of body temperature, with or without sedation, on energy expenditure in severe head-injured patients. Crit Care Med. (1998) 26:568–72. 10.1097/00003246-199803000-00033 9504588

[22] Bruder N Lassegue D Pelissier D Graziani N François G. Energy expenditure and withdrawal of sedation in severe head-injured patients. Crit Care Med. (1994) 22:1114–9. 10.1097/00003246-199407000-00011 8026200

[23] Clifton GL Robertson CS Choi SC. Assessment of nutritional requirements of head-injured patients. J Neurosurg. (1986). 64:895–901. 10.3171/jns.1986.64.6.0895 3701439

[24] Dempsey DT Guenter P Mullen JL Fairman R Crosby LO Spielman G. Energy expenditure in acute trauma to the head with and without barbiturate therapy. Surg Gynecol Obstet. (1985) 160:128–34. 3918351

[25] Matthews DS Aynsley-Green A Matthews JN Bullock RE Cooper BG Eyre JA. The effect of severe head injury on whole body energy expenditure and its possible hormonal mediators in children. Pediatr Res. (1995) 37:409–17. 10.1203/00006450-199504000-00005 7596679

[26] Havalad S Quaid M Sapiega V. Energy Expenditure in Children With Severe Head Injury: Lack of Agreement Between Measured and Estimated Energy Expenditure. Nutr Clin Pract. (2006) 21:175–81. 10.1177/0115426506021002175 16556928

[27] Mtaweh H Smith R Kochanek PM Stephen R Fabio A Vavilala MS. Energy expenditure in children after severe traumatic brain injury. Pediatric Crit Care Med. (2014) 15:242–9. 10.1097/PCC.0000000000000041 24394999 PMC4703075

[28] Skillman HE. Enteral Nutrition in the Critically ill Child. In: Goday PS Mehta NM editors. Pediatric Critical Care Nutrition. New York, NY: McGraw-Hill Education (2015).

[29] Kelly DA. Liver complications of pediatric parenteral nutrition-epidemiology. Nutrition. (1998) 14:153–7. 10.1016/S0899-9007(97)00232-3 9437702

[30] Fivez T Kerklaan D Mesotten D Verbruggen S Wouters PJ Vanhorebeek I. Early versus late parenteral nutrition in critically ill children. New England J Med. (2016) 374:1111–22. 10.1056/NEJMoa1514762 26975590

[31] Justo Meirelles CM de Aguilar-Nascimento JE. Enteral or parenteral nutrition in traumatic brain injury: a prospective randomised trial. Nutr Hospitalaria. (2011) 26:1120–4. 10.1590/S0212-16112011000500030 22072362

[32] Mehta NM Bechard LJ Zurakowski D Duggan CP Heyland DK. Adequate enteral protein intake is inversely associated with 60-d mortality in critically ill children: a multicenter, prospective, cohort study. Am J Clin Nutr. (2015) 102:199–206. 10.3945/ajcn.114.104893 25971721 PMC4480666

[33] Mehta NM McAleer D Hamilton S Naples E Leavitt K Mitchell P. Challenges to optimal enteral nutrition in a multidisciplinary pediatric intensive care unit. J Parenteral Enteral Nutr. (2010) 34:38–45. 10.1177/0148607109348065 19903872 PMC4902117

[34] de Oliveira Iglesias S Leite H Santana e Meneses JF de Carvalho W. Enteral nutrition in critically ill children: are prescription and delivery according to their energy requirements? Nutr Clin Pract. (2007) 22:233–9. 10.1177/0115426507022002233 17374797

[35] Panchal AK Manzi J Connolly S Christensen M Wakeham M Goday PS. Safety of enteral feedings in critically ill children receiving vasoactive agents. J Parenteral Enteral Nutr. (2016) 40:236–41. 10.1177/0148607114546533 25168592

[36] Pinto TF Rocha R Paula CA de Jesus RP. Tolerance to enteral nutrition therapy in traumatic brain injury patients. Brain Injury. (2012) 26:1113–7. 10.3109/02699052.2012.666369 22571511

[37] Härtl R Gerber LM Ni Q Ghajar J. Effect of early nutrition on deaths due to severe traumatic brain injury. J Neurosurg. (2008) 109:50–6. 10.3171/JNS/2008/109/7/0050 18590432

[38] Taha AA Badr L Westlake C Dee V Mudit M Tiras KL. Effect of early nutritional support on intensive care unit length of stay and neurological status at discharge in children with severe traumatic brain injury. J Neurosci Nurs. (2011) 43:291–7. 10.1097/JNN.0b013e318234e9b2 22089405

[39] Meinert E Bell M Buttram S Kockanek P Balasubramani G Wisniewski S. Initiating nutritional support before 72 hours is associated with favorable outcome after severe traumatic brain injury in children: a secondary analysis of a randomized, controlled trial of therapeutic hypothermia. Pediatr Crit Care Med. (2018) 19:345–52. 10.1097/PCC.0000000000001471 29370008 PMC5886794

[40] Balakrishnan B. Flynn-O'Brien KT, Simpson PM, Dasgupta M, Hanson SJ. Enteral nutrition initiation in children admitted to pediatric intensive care units after traumatic brain injury. Neurocr Care. (2019) 30:193–200. 10.1007/s12028-018-0597-6 30171446

[41] Briassoulis G Filippou O Kanariou M Papassotiriou I Hatzis T. Temporal nutritional and inflammatory changes in children with severe head injury fed a regular or an immune-enhancing diet: a randomized, controlled trial. Pediatric Critical Care Medicine. (2006) 7:56–62. 10.1097/01.PCC.0000192339.44871.26 16395076

[42] Meert KL Daphtary KM Metheny NA. Gastric vs small-bowel feeding in critically ill children receiving mechanical ventilation: a randomized controlled trial. Chest. (2004) 126:872–8. 10.1378/chest.126.3.872 15364769

[43] Ott L Young B Phillips R Mcclain C Adams L Dempsey R Tibbs P Ryo UY. Altered gastric emptying in the head-injured patient: relationship to feeding intolerance. J Neurosurg. (1991) 74:738–742. 10.3171/jns.1991.74.5.0738 1901599

[44] Rhoney DH Parker D Formea CM Yap C Coplin WM. Tolerability of bolus versus continuous gastric feeding in brain-injured patients. Neurol Res. (2002) 24:613–20. 10.1179/016164102101200456 12238631

[45] Heyland DK Novak F Drover JW Jain M Su X. Suchner U. Should immunonutrition become routine in critically ill patients? A systematic review of the evidence. JAMA. (2001) 286:944–53. 10.1001/jama.286.8.944 11509059

[46] Simon DW McGeachy MJ Baylr H Clark RSB Loane DJ Kochanek PM. The far-reaching scope of neuroinflammation after traumatic brain injury. Nat Rev Neurol. (2017) 13:171–91. 10.1038/nrneurol.2017.13 28186177 PMC5675525

[47] Montejo JC Zarazaga A López-Martínez J Urrútia G Roqué M Blesa AL. Immunonutrition in the intensive care unit A systematic review and consensus statement. Clin Nutr. (2003) 22:221–33. 10.1016/S0261-5614(03)00007-4 12765660

[48] Hanscom M Loane DJ Shea-Donohue T. Brain-gut axis dysfunction in the pathogenesis of traumatic brain injury. J Clin Investig. (2021) 131:e143777. 10.1172/JCI143777 34128471 PMC8203445

[49] Rogers MB Simon D Firek B Silfies L Fabio A Bell M. Temporal and spatial changes in the microbiome following pediatric severe traumatic brain injury. Pediat Crit Care Med. (2022). 10.1097/PCC.0000000000002929. [Epub ahead of print]. 35283451 PMC9203870

[50] Mechanick JI. Metabolic mechanisms of stress hyperglycemia. J Parenter Enteral Nutr. (2006) 30:157–163 10.1177/0148607106030002157 16517960

[51] Lam AM Ricttard Winn H Cullen BE Sundling N. Hyperglycemia and neurological outcome in patients with head injury. J Neurosurg. (1991). 75:545–551. 10.3171/jns.1991.75.4.0545 1885972

[52] Srinivasan V. Stress hyperglycemia in pediatric critical illness: the intensive care unit adds to the stress! J Diab Sci Technol. (2012) 6:37–47. 10.1177/193229681200600106 22401321 PMC3320820

[53] Chen S Liu Z. Effect of hyperglycemia on all-cause mortality from pediatric brain injury: a systematic review and meta-analysis. Medicine. (2020) 99:e23307. 10.1097/MD.0000000000023307 33235087 PMC7710234

[54] Saadat SMS Bidabadi E Saadat SNS Mashouf M Salamat F Yousefzadeh S. Association of persistent hyperglycemia with outcome of severe traumatic brain injury in pediatric population. Child's Nerv Syst. (2012) 28:1773–7. 10.1007/s00381-012-1753-5 22526446

[55] Smith RL Lin JC Adelson PD Kochanek PM Fink EL Wisniewski SR. Relationship between hyperglycemia and outcome in children with severe traumatic brain injury. Pediat Crit Care Med. (2012) 13:85–91. 10.1097/PCC.0b013e3182192c30 21499170 PMC3677026

[56] Jeremitsky E Omert L Dunham C Wilberger J Rodriguez A. The impact of hyperglycemia on patients with severe brain injury. J Trauma. (2005) 58:47–50. 10.1097/01.TA.0000135158.42242.B1 15674149

[57] Agus MSD Wypij D Hirshberg EL Srinivasan V Faustino EV Luckett PM. Tight glycemic control in critically ill children. New England J Med. (2017) 376:729–41. 10.1056/NEJMoa1612348 28118549 PMC5444653

[58] Macrae D Grieve R Allen E Sadique Z Morris K Pappachan J. Randomized trial of hyperglycemic control in pediatric intensive care. New England J Med. (2014) 370:107–18. 10.1056/NEJMoa1302564 24401049

[59] Agus MSD Steil GM Wypij D Costello JM Laussen PC Langer M. Tight glycemic control versus standard care after pediatric cardiac surgery. New England J Med. (2012) 367:1208–19. 10.1056/NEJMoa1206044 22957521 PMC3501680

[60] Finfer S Chittock D Li Y Foster D Dhingra V Bellomo R. Intensive versus conventional glucose control in critically ill patients with traumatic brain injury: long-term follow-up of a subgroup of patients from the NICE-SUGAR study. Intensive Care Med. (2015) 41:1037–47. 10.1007/s00134-015-3757-6 26088909

[61] Vespa P Boonyaputthikul R McArthur DL Miller C Etchepare M Bergsneider M. Intensive insulin therapy reduces microdialysis glucose values without altering glucose utilization or improving the lactate/pyruvate ratio after traumatic brain injury. Crit Care Med. (2006) 34:850–6. 10.1097/01.CCM.0000201875.12245.6F 16505665

[62] Vespa P McArthur DL Stein N Huang SC Shao W Filippou M. Tight glycemic control increases metabolic distress in traumatic brain injury: a randomized controlled within-subjects trial. Crit Care Med. (2012) 40:1923–9. 10.1097/CCM.0b013e31824e0fcc 22610193

[63] Venter C Eyerich S Sarin T Klatt KC. Nutrition and the immune system: A complicated tango. Nutrients. (2020) 12:818. 10.3390/nu12030818 32204518 PMC7146186

[64] Johnson RW Ng KWP Dietz AR Hartman ME Baty JD Hasan N. Muscle atrophy in mechanically-ventilated critically ill children. PLoS ONE. (2018) 13:e0207720. 10.1371/journal.pone.0207720 30566470 PMC6300323

[65] Briassoulis G Zavras N Hatzis T. Malnutrition, nutritional indices, and early enteral feeding in critically ill children. Nutrition. (2001) 17:548–57. 10.1016/S0899-9007(01)00578-0 11448572

[66] Cook A Peppard A Magnuson B. Nutrition considerations in traumatic brain injury. Nutr Clin Pract. (2008) 23:608–20. 10.1177/0884533608326060 19033220

[67] Bochicchio G Bochicchio K Nehman S Casey C Andrews P Scalea T. Tolerance and efficacy of enteral nutrition in traumatic brain-injured patients induced into barbiturate coma. JPEN J Parenter Enteral Nutr. (2006) 30:503–6. 10.1177/0148607106030006503 17047175

[68] Malakouti A Sookplung P Siriussawakul A Philip S Bailey N Brown M. Nutrition support and deficiencies in children with severe traumatic brain injury. Pediatr Crit Care Med. (2012) 13:e18–24. 10.1097/PCC.0b013e31820aba1f 21317678

[69] Kochanek PM Tasker RC Carney N Totten AM Adelson PD Selden NR. Guidelines for the management of pediatric severe traumatic brain injury, third edition: Update of the brain trauma foundation guidelines. Pediat Crit Care Med. (2019). 20:1–82. 10.1097/PCC.0000000000001735 30829890

[70] Pollack MM Holubkov R Glass P Dean JM Meert KL Zimmerman J. Functional status scale: new pediatric outcome measure. Pediatrics. (2009) 124:e18-28. 10.1542/peds.2008-1987 19564265 PMC3191069

